# Supplementary material for: Diagnosing Middle Ear Malformation by Pure-Tone Audiometry Using a Three-Dimensional Finite Element Model: A Case-Control Study
Source: J Clin Med. 2023 Dec 4;12(23):7493. doi: 10.3390/jcm12237493 (PMC10707247; doi:10.3390/jcm12237493)
Supplement: Supplementary file 1 [file jcm-12-07493-s001.zip › jcm-2699282-supplementary.pptx]

## Slide 1
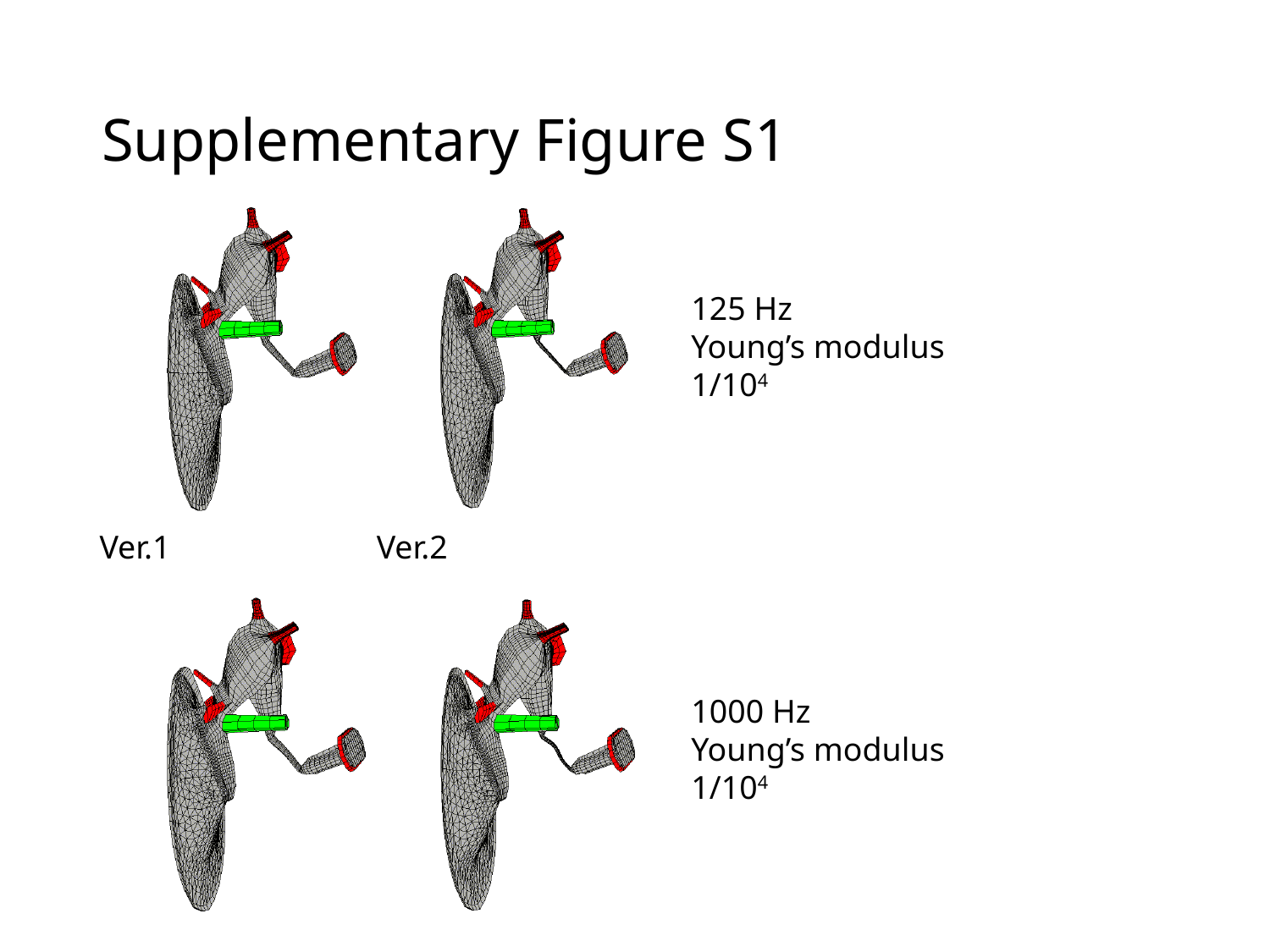

# Supplementary Figure S1
125 Hz
Young’s modulus 1/104
Ver.1
Ver.2
1000 Hz
Young’s modulus 1/104

## Slide 2
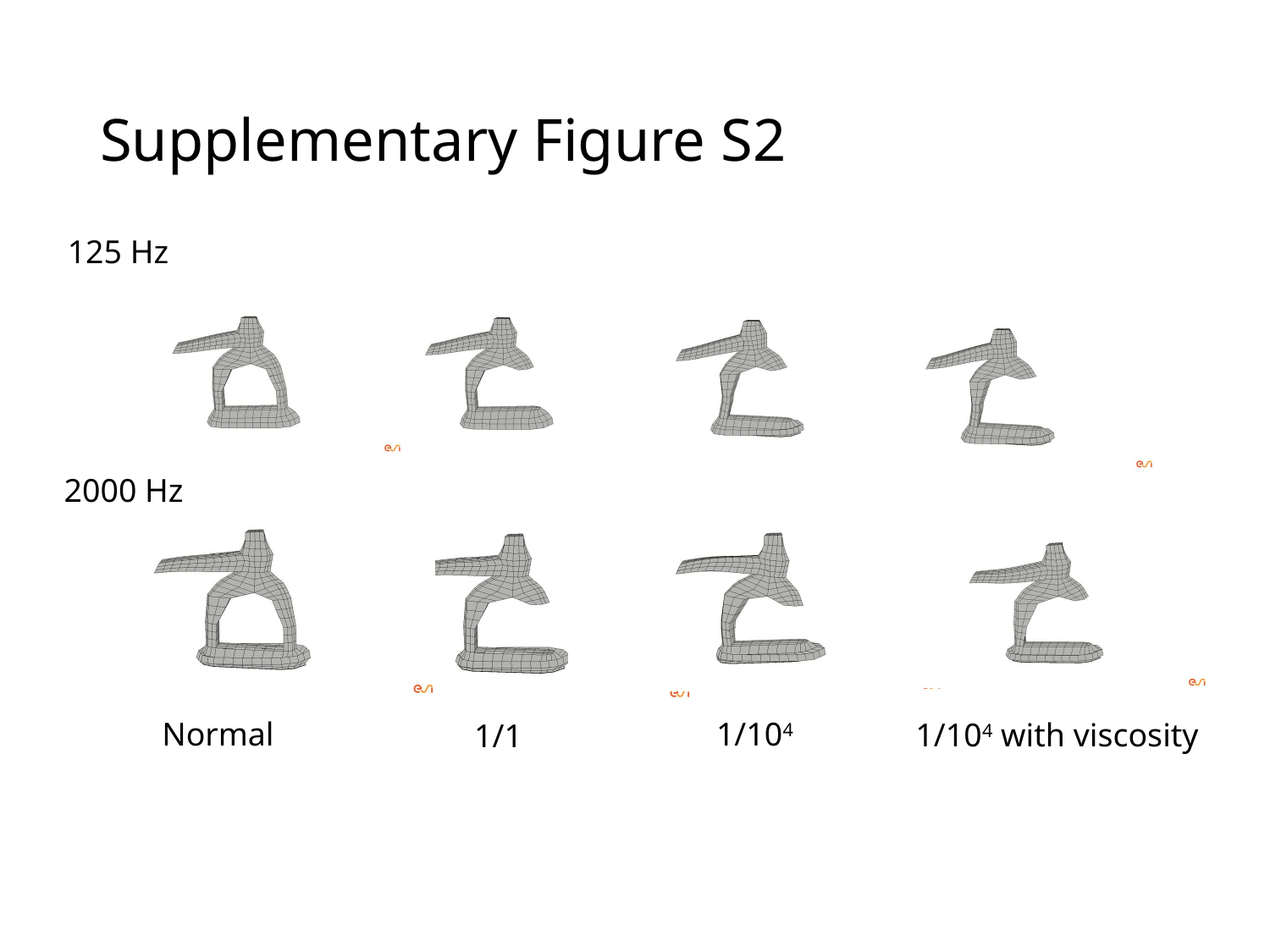

# Supplementary Figure S2
125 Hz
2000 Hz
Normal
1/104
1/104 with viscosity
1/1

## Slide 3
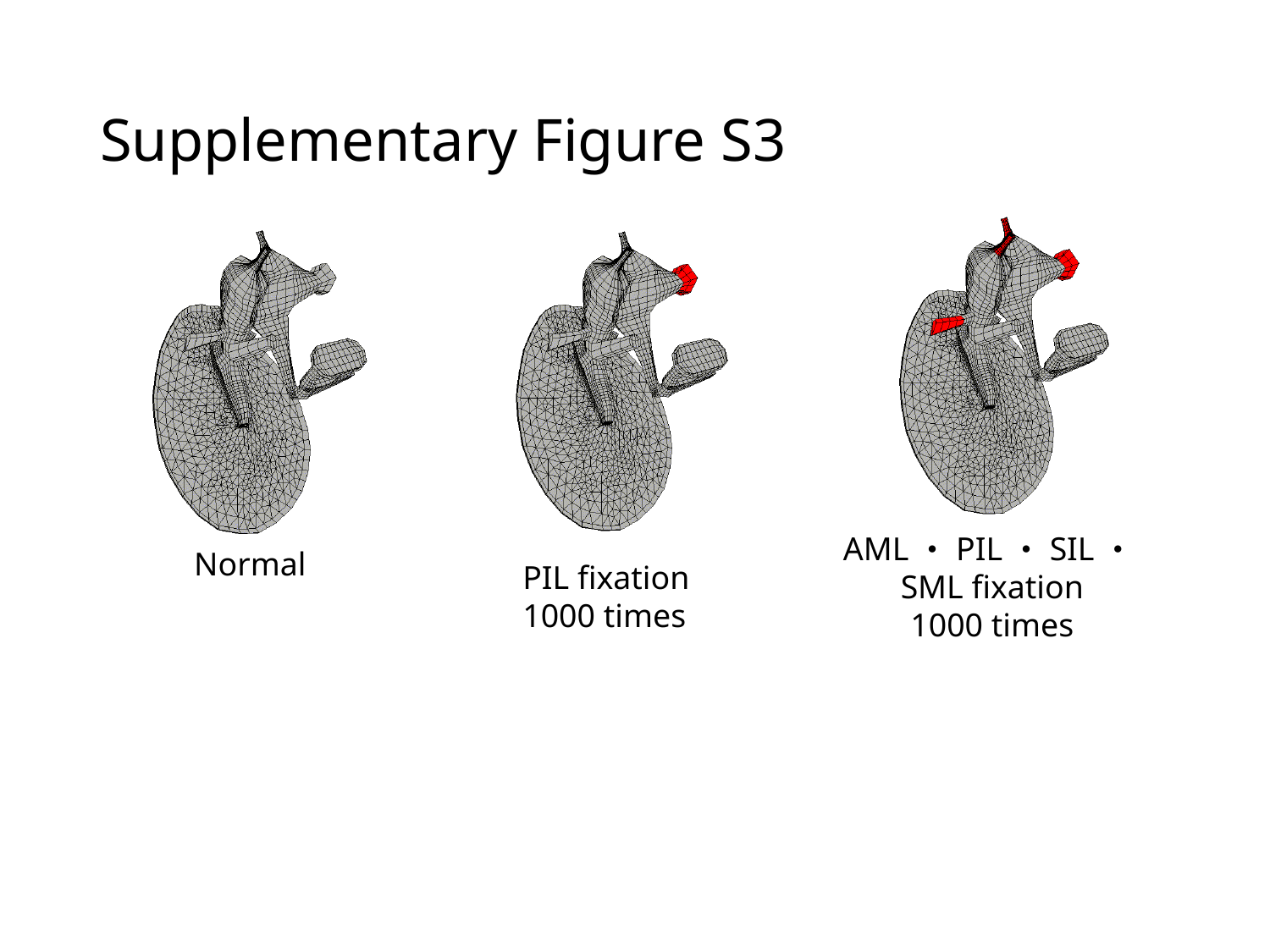

# Supplementary Figure S3
AML・PIL・SIL・SML fixation
1000 times
Normal
PIL fixation
1000 times
